# Treatment and outcomes of immunotherapy related colitis and hepatitis- a multi-centre cohort study in the United Kingdom by the National Oncology Trainee Collaborative for Healthcare Research (NOTCH)

**DOI:** 10.1007/s00520-026-10794-9

**Published:** 2026-05-29

**Authors:** Mirashini Swaminathan, Angelos Angelakas, Mark Baxter, Jenny Cotton, Caroline Dobeson, Laura Feeney, Abigail Gault, Daniel J. Hughes, Chris Jones, R. Lee, Sohail Mughal, Shefali Parikh, David Mark Pritchard, Lisa Rodgers, Michael Rowe, Abdulazeez Salawu, Rohan Shotton, Nadina Tinsley, Ann Tivey, Steven Zhao, Anna Claire Olsson-Brown

**Affiliations:** 1 The Wolfson Centre for Personalised Medicine, University of Liverpool, Block A: Waterhouse Buildings, 1-5 Brownlow Street, Liverpool, L69 3GL UK; 2 Princess Margaret Cancer Centre, University Health Network, Toronto, ON Canada; 3 Tayside Cancer Centre, Dundee, UK; 4 Clatterbridge Cancer Centre, Liverpool, UK; 5 Northern Centre for Cancer Care, Newcastle-Upon-Tyne, UK; 6 Christie Hospital, Manchester, UK; 7 Kings College London, London, UK; 8 Leeds Cancer Centre, Leeds, UK; 9 Newcastle University, Newcastle-Upon-Tyne, UK; 10 Bradford Teaching Hospitals NHS Foundation Trust, Leeds, UK; 11 Beaston West of Scotland Cancer Centre, Glasgow, UK; 12 Royal Cornwall Hospitals NHS Trust, Truro, UK; 13 University Hospitals Sussex, Brighton, UK

**Keywords:** Immune checkpoint inhibitors, Immune related adverse events, Gastrointestinal immune related adverse events, Immune checkpoint inhibitor related colitis, Immune checkpoint inhibitor related hepatitis

## Abstract

**Purpose:**

Immune checkpoint inhibitors (ICIs) are increasingly used in the treatment of many cancers but are associated with immune-related adverse events (irAEs), including gastrointestinal toxicities such as colitis and hepatitis. This study aimed to evaluate the incidence of GI-irAEs, identify risk factors for their development, and assess the relationship between GI-irAEs, immunosuppressive treatment, and overall survival (OS) in a large real-world cohort.

**Methods:**

A multi-centre retrospective study was conducted across 12 National Health Service centres in the United Kingdom between June 2016 and 2018. Consecutive adult patients receiving ICIs for malignant melanoma, non-small cell lung cancer, or renal cell carcinoma were included. Clinically significant (≥ grade 2) GI-irAEs were recorded. Logistic regression was used to identify predictive factors for GI-irAEs and immunosuppressant (IS) use. OS was assessed using Kaplan–Meier survival analysis and log-rank testing.

**Results:**

A total of 2,049 patients were included, of whom 1,649 were included in the GI-irAE subgroup analysis. Colitis occurred in 191 (9.3%) patients and hepatitis in 142 (6.9%). The majority of patients were treated with steroids alone; 22.3% of colitis cases and 15.8% of hepatitis cases required second line IS. Colitis and hepatitis were both associated with improved OS compared to patients without irAEs (*p* < 0.0001). Pre-existing autoimmune disease and combination immunotherapy predicted IS use in colitis, while grade 2–3 hepatitis predicted IS use. IS did not significantly affect OS in colitis but was associated with improved OS in hepatitis.

**Conclusion:**

Colitis and hepatitis are common GI-irAEs following ICI therapy and are associated with improved OS. The use of IS does not negatively impact survival, supporting their continued use in the management of steroid-refractory GI-irAEs and informing supportive care strategies for risk stratification and optimisation of toxicity management in patients receiving immunotherapy.

## Introduction

Immune checkpoint inhibitors (ICIs) have revolutionised the treatment of many cancers over the last decade. They function by inhibiting checkpoint proteins that are responsible for inducing the exhaustion of T-lymphocytes, thereby activating immune cells to target tumour antigens [1]. ICIs utilised in current clinical practice include those targeted against the checkpoint proteins cytotoxic T-lymphocyte antigen-4 (CTLA-4), programmed death protein-1 (PD-1) and programmed death ligand-1 (PD-L1). Following the impressive early responses to ICIs seen in melanoma, their use—either alone or in combination with more conventional cancer treatments—has expanded to encompass a wide variety of cancers in the settings of locally advanced and metastatic disease.

It became clear during early phase clinical trials involving these agents that ICIs are associated with unique adverse events known as immune related adverse events (irAEs). More than half of patients receiving ICI therapy develop irAEs of varying severity and these can have significant impacts on mortality and morbidity [2, 3]. Studies have shown that while patients with irAEs may experience improved progression-free survival, it is crucial to balance this efficacy with the potential for treatment disruption and the impact on patient quality of life [4]. These are thought to develop as a consequence of the dysregulation of the immune system that results from inhibition of immune checkpoint proteins [5]. IrAEs can affect any organ in the body, but are most commonly observed in the skin, bowel, liver and endocrine organs [5]. Gastrointestinal toxicities (GI-irAEs) in the form of colitis and hepatitis represent the second and third most common irAEs following dermatitis [6].

ICI-induced GI-irAEs most commonly occur in the colon resulting in colitis; however, inflammation of the oesophagus, stomach and small bowel have also been reported. The descending colon is the most commonly affected site for colitis, which most frequently occurs following anti–CTLA-4 and anti–PD-1/PD-L1 blockade [7]. It is one of the most common causes of discontinuation of ICI therapy and treatment-related death [8].

Colitis is diagnosed and graded according to the Common Terminology Criteria for Adverse Events (CTCAE) criteria (see Table 1) [9]. Patients with grade 1 symptoms are often managed conservatively and investigated for alternative causes such as infection. If symptoms persist or escalate to grade 2, oral steroid therapy is often commenced. At this point, patients may be referred for a flexible sigmoidoscopy to assess for the presence of inflammation. Severe colitis (grade 3 or 4) may rarely lead to life-threatening complications such as colonic perforation and usually requires treatment with intravenous steroids. Patients who do not respond to this therapy after 3–5 days are considered to be steroid refractory and second line immunosuppressants (IS) such as the anti-tumour necrosis factor-alpha inhibitor (anti-TNF), infliximab are used.

**Table 1:** Grading of colitis and hepatitis as defined by the CTCAE criteria

Adverse event	Grade 1	Grade 2	Grade 3	Grade 4	Grade 5
Colitis	Asymptomatic; clinical or diagnostic observations only: intervention not indicated	Abdominal pain; mucous or blood in stool	Severe abdominal pain change in bowel habits; medical intervention indicated; peritoneal signs	Life-threatening consequences; urgent intervention indicated	Death
Hepatitis	AST or ALT 1- 2.5 X ULN and/or T-BIL 1–1.5 X ULN	AST or ALT 2–5 X ULN and/or T-BIL 1.5–3 X ULN	AST or ALT > 5 X ULN and or T-BIL > 3 X ULN	AST or ALT > 8 X ULN	Death

Abbreviations: CTCAE, Common Terminology for Cancer Adverse Events; AST, aspartate transaminase; ALT, alanine transaminase, ULN, upper limit of normal; T-BIL, total bilirubin

As with colitis, the incidence of hepatitis is higher in patients receiving combination ICI therapy. Generally, the incidence is reported to be around 2% to 10% in single agent ICI therapy and up to 40% in combination therapy [10]. Patients who develop hepatitis secondary to ICIs are often asymptomatic or have non-specific symptoms such as fatigue and malaise. Hepatitis is most commonly identified during routine blood tests. It usually manifests as a transaminitis with elevation of aspartate aminotransferase (AST) and alanine aminotransferase (ALT). Hyperbilirubinemia is rarely a feature but can sometimes be seen giving a cholestatic picture. Grading of hepatitis defined by the CTCAE criteria is based on the degree of elevation of AST and ALT and the presence or absence of hyperbilirubinemia (see Table 1). Hepatitis responds well to treatment with steroids, but resolution can take up to 12 weeks [11]. In steroid refractory cases, Mycophenolate Mofetil (MMF) or Tacrolimus can be used.

As real-world patients often differ from trial populations in terms of age and comorbidity, the extent to which the real-world incidence of these GI-irAEs mirrors that seen in clinical trials is uncertain. There is also a need to understand whether there are predictive factors for the development of GI-irAEs, how the development of GI-irAEs impacts on overall survival and how in particular the use of immunosuppressive treatments to manage GI-irAEs impacts on patient outcomes. We sought to evaluate these aspects within a multi-centre study focussed on a large real-world population of patients diagnosed with metastatic melanoma (MM), non-small cell lung cancer (NSCLC) and renal cell cancer (RCC).

## Methods

### Approach

A retrospective observational study was carried out by examining patient records across 12 cancer centres in the United Kingdom (UK) between June 2016 and June 2018. Data collection was overseen and undertaken by The UK National Oncology Trainees Collaborative for Healthcare Research (NOTCH) [12]. Audit approvals were gained in each centre and Caldicott approvals were secured for transfer of anonymised data.

### Inclusion and exclusion criteria

Consecutive patients aged ≥ 18 years who started ICI treatment for metastatic or locally advanced MM, NSCLC and RCC within the National Health Service between 1 st June 2016 and 1 st June 2018 were included in the study with a censor date of 1 st November 2018. Only patients treated with ICIs or combination ICIs were included. Patients who received ICIs in combination with other systemic anti-cancer therapies (SACT) or other targeted therapies were excluded. This was to ensure that true irAEs related to ICI treatment only were captured.

### Data collection

Patients were identified using electronic patient records and data were collected using a standardised pre-piloted data collection tool (Excel 2013). Extracted data items included patient characteristics (age, gender, smoking history, presence or absence of a pre-existing autoimmune condition), cancer type and details of ICI treatment. Details of clinically significant (CTCAE grade 2 or above) irAEs were also recorded. All instances of documented irAEs, as diagnosed by each patient’s treating team in accordance with local guidance, were recorded. These were graded as per contemporaneous clinician grading or, if this was not available, retrospectively in accordance with criteria within CTCAE version 5.

### Data analysis

Data were summarised using frequencies, percentages, medians and ranges. Statistical significance was determined using t-tests for normally distributed data. Mann–Whitney analysis was used for non-normally distributed data, and Chi-squared and Fisher’s exact test used for categorical data. All analyses were performed using GraphPad Prism version 9. Multiple logistic regression was used to determine risk factors for GI-irAEs. Overall survival (OS) was estimated using Kaplan–Meier methodology with statistical significance determined by using the log-rank test. A *p* value of < 0.05 was considered statistically significant.

## Results

### Patient characteristics

A total of 2,049 patients across 12 sites were eligible for inclusion and were followed up until the censor date specified. Of these, 1,649 patients were included in the comparative analysis presented in Table 2, comprising those who developed colitis (*n* = 191), hepatitis (*n* = 142), or no irAEs (*n* = 1,316). The remaining 400 patients experienced non-gastrointestinal irAEs and were not included in this subgroup analysis. Demographic data for the 1,649 patients are summarised in Table 2. Of these, 980 (59.4%) were male and the median age was 66 years (range 23–91). A total of 726 (44.0%) patients had metastatic melanoma, 638 (38.7%) had non-small-cell lung cancer, and 285 (17.3%) had renal cell carcinoma. The majority of patients (1,427; 86.5%) received ICI monotherapy (mICI), while 222 (13.5%) received combination αCTLA-4/αPD-1 therapy (cICI), which was only used in the melanoma group. These treatment patterns were consistent with standard practice at the time of the study.

**Table 2:** Patient characteristics

Characteristics		N (%)			*p*-value
		Colitis	Hepatitis	No irAEs	
Cohort size		191	142	1316	
Age starting ICI- Median (IQR)					< 0.0001*
		64 (56–71)	60 (48–67)	67 (58–73)	
Gender					0.25
	Male	111 (58.1)	94 (66.2)	775 (58.9)	
	Female	80 (41.9)	48 (33.8)	541 (41.1)	
Smoking status					0.24
	Never	66 (34.6)	48 (33.8)	327 (24.8)	
	Ex	57 (29.8)	45 (31.7)	468 (35.6)	
	Current	22 (11.5)	18 (12.7)	204 (15.5)	
	Unknown	46 (24.1)	30 (21.3)	317 (24.1)	
Malignancy type					< 0.0001*
	Metastatic melanoma	131 (68.6)	108 (76.1)	487 (37)	
	Non-small cell lung cancer	43 (22.5)	28 (19.7)	567 (43.1)	
	Renal cell carcinoma	17 (8.9)	6 (4.2)	262 (19.9)	
Pre-existing autoimmune disease (PAID)					< 0.001*
	Yes	41 (21.5)	15 (10.6)	134 (10.2)	
	No	150 (78.5)	126 (88.7)	1182 (89.8)	
ICI drug					< 0.0001*
	Ipilimumab/Nivolumab	85 (44.5)	69 (48.6)	68 (5.2)	
	Ipilimumab	9 (4.7)	3 (2.1)	22 (1.7)	
	Nivolumab	21 (11)	12 (8.5)	358 (27.2)	
	Pembrolizumab	76 (39.8)	58 (40.8)	852 (64.7)	
	Atezolizumab	0	0	16 (1.2)	

^*^denotes statistical significance at *p* < 0.05

### Incidence of GI-irAEs

Within the overall cohort, a total of 733 (35.8%) patients experienced any clinically significant (grade ≥ 2) irAE. Three hundred thirty-seven (16.4%) patients developed GI-irAE, 191 (9.3%) colitis and 142 (6.9%) hepatitis. Amongst the grade ≥ 2 irAEs, colitis was the most common irAE and hepatitis was the third most common irAE experienced.

One hundred fifty-nine (83.2%) of those patients who developed colitis experienced it as a first toxicity, whereas 93 (65.5%) of patients who developed hepatitis experienced it as their first toxicity. The median time from initiation of ICI to developing colitis and hepatitis was 2 and 1.7 months respectively. The relative incidences of the various toxicities are displayed in Table 3.

**Table 3:** Incidence of first toxicities in the overall cohort

1 st Toxicity	N	%
Total	733	35.8
Colitis	159	7.8
Dermatitis	125	17.1
Thyroid disorder	103	5
Hepatitis	93	4.5
Pneumonitis	52	2.5
Arthritis	35	1.7
Unknown	34	1.7
Other	31	1.5
Nephritis	28	1.4
Hypophysitis	24	1.2
Neuropathy	15	0.7
Uveitis	14	0.7
Diabetes	10	0.5
Haematological	5	0.2
Myositis	4	0.2
Pancreatitis	1	0.1

Of the patients who experienced colitis, 103 (53.9%) were graded as severe and there were 84 (59.2%) incidences of severe hepatitis (defined as ≥ grade 3). Colitis was the most commonly occurring severe irAE in the overall cohort with hepatitis being the second most common. No deaths (grade 5) were recorded in either group.

### Risk factors predicting the development of GI-irAEs

Multiple logistic regression analysis was undertaken to determine whether there were any risk factors that could predict the development of GI-irAEs (Table 4). The presence of any pre-existing autoimmune diseases (PAID) (HR: 1.86, 95%CI: 1.25–2.78, *p* = 0.002) and the type of ICI therapy (cICI vs mICI) (HR: 7.20, 95%CI: 4.74–10.95, *p* < 0.0001) were significant predictors for the occurrence of colitis. Cancer type was associated with the development of hepatitis, with patients with non-melanoma malignancies having lower odds compared to those with melanoma (HR 0.54, 95% CI 0.38–0.78, *p* < 0.001), indicating a higher risk in the melanoma group. Combination ICI therapy (HR 4.19, 95% CI 2.71–6.47, *p* < 0.001) and younger age (HR 0.98, 95% CI 0.96–0.99, *p* < 0.001) were also independent predictors of hepatitis. Due to cICI only being utilised in MM as this was only available to patients with MM at the time of the study, the analysis was repeated for mICI alone and those predictors remained significant. Seventeen (0.8%) patients had pre-existing inflammatory bowel disease (IBD) in the cohort and 6 of them subsequently developed colitis (35%). In patients without IBD, the rate of colitis was 185/2032 (9.1%).

**Table 4:** Multivariate logistic regression for risk of GI-irAEs

Characteristic	Colitis		Hepatitis	
	**OR (95% CI)**	***p*-value**	**OR (95% CI)**	***p*-value**
Age	1.01 (0.99–1.02)	0.185	0.98 (0.96–0.99)	< 0.001*
Gender (Male)	1.14 (0.831–1.58)	0.409	0.768 (0.52–1.11)	0.157
Smoking status	0.95 (0.83–1.09)	0.476	0.93 (0.80–1.08)	0.342
Malignancy (Melanoma)	0.85 (0.64–1.13)	0.259	0.54 (0.38–0.78)	< 0.001*
Number of metastatic sites	0.93 (0.80–1.07)	0.312	1.02 (0.87–1.21)	0.797
PAID	1.86 (1.25–2.78)	0.002*	0.314 (0.42–1.33)	0.314
ICI therapy (cICI vs mICI)	7.20 (4.74–10.95)	< 0.001*	4.19 (2.71–6.47)	< 0.001*

^*^denotes statistical significance at *p* < 0.05

### Overall survival in patients with GI-irAEs

**Fig. 1:**
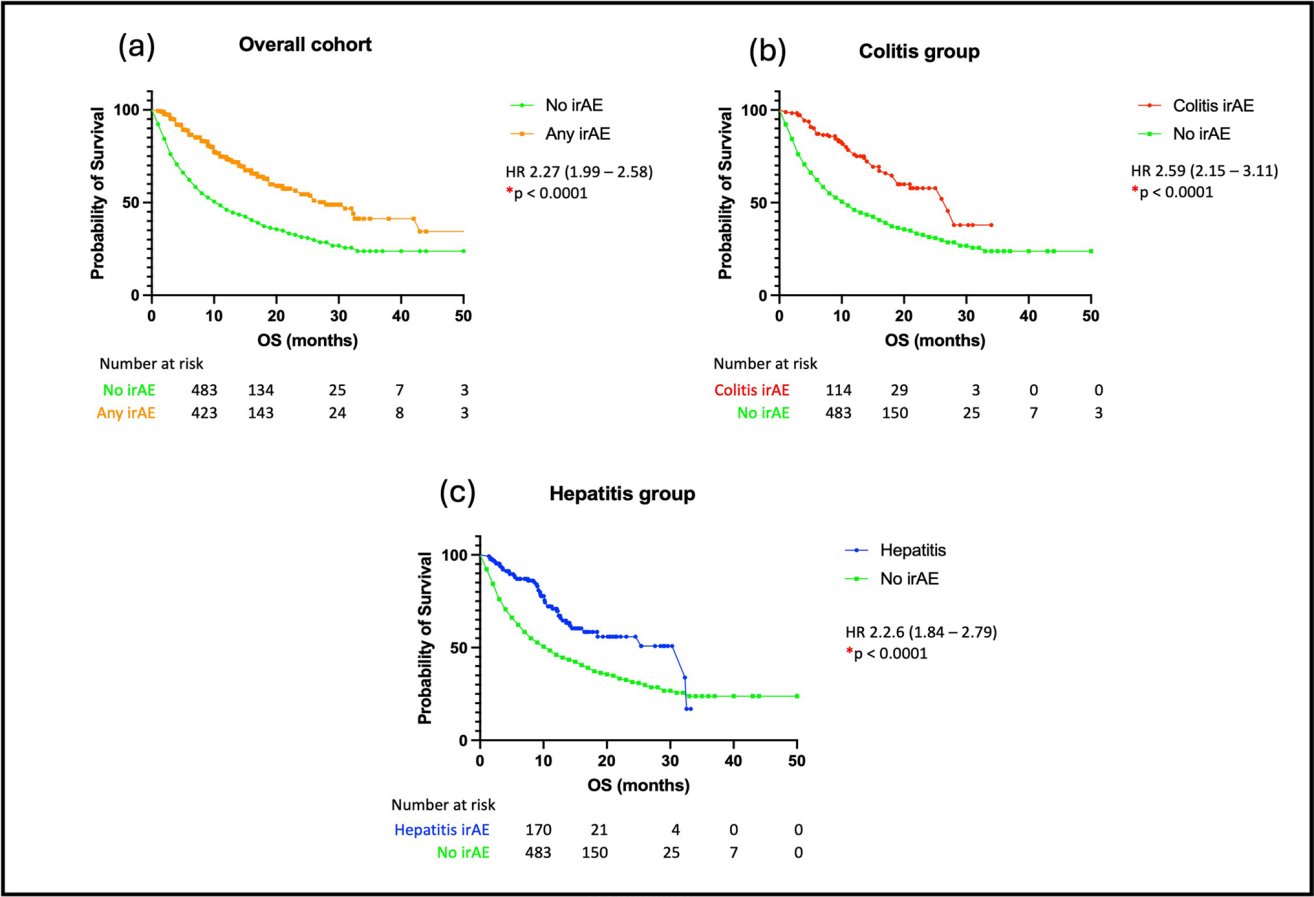

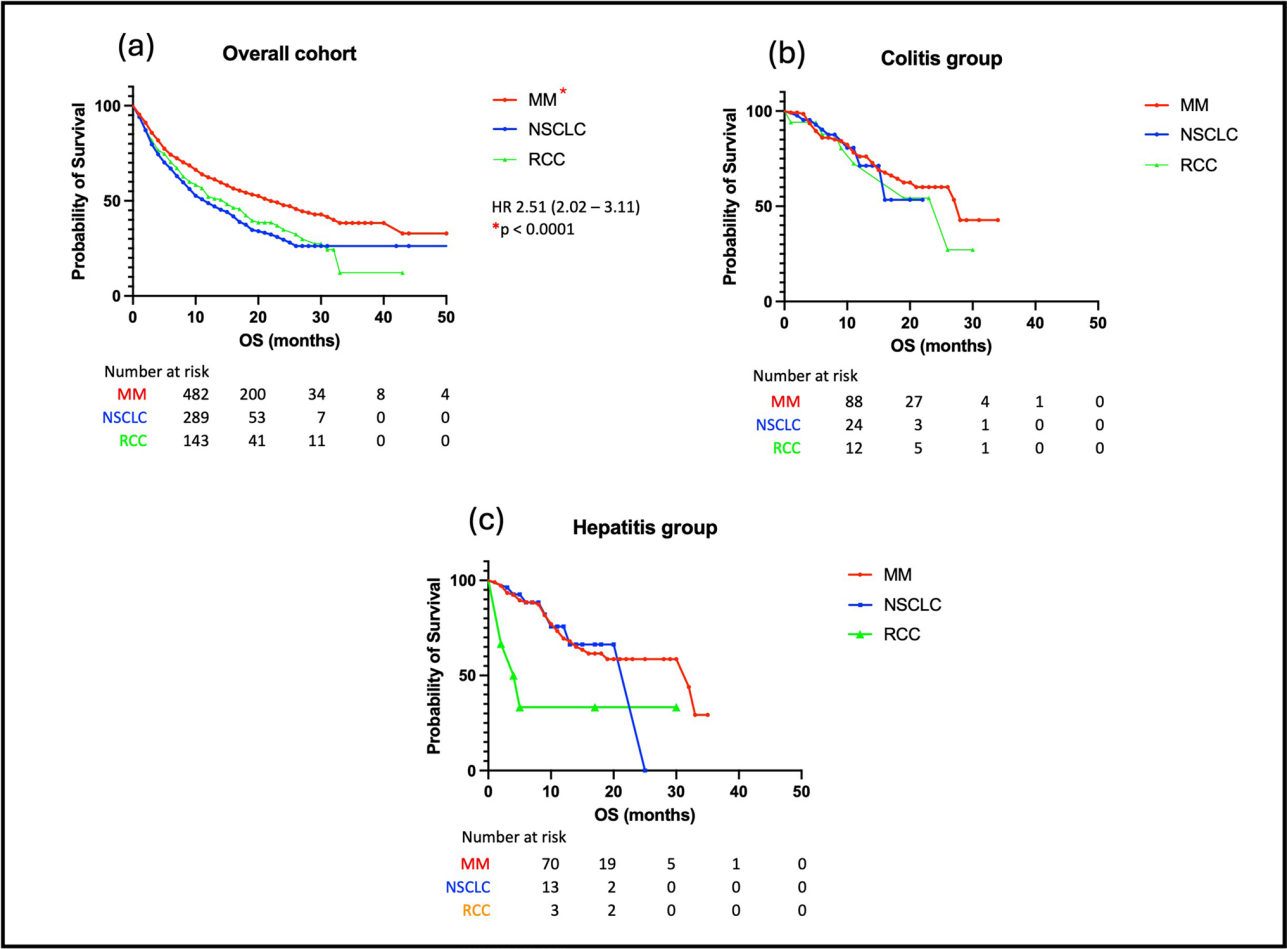
**A** Overall survival (OS) of (**a**) Patients with any immune related adverse event (irAE) in the overall cohort compared to patients with no irAEs; (**b**) Patients with colitis compared to patients with no irAEs and (**c**) Patients with hepatitis compared to patients with no irAEs. **B** Overall survival (OS) of patients based on malignancy subtype- Metastatic melanoma (MM), non-small cell lung cancer (NSCLC) and renal cell cancer (RCC) (**a**) Patients in the overall cohort; (**b**) Patients with colitis and (**c**) Patients with hepatitis

**Fig. 2:**
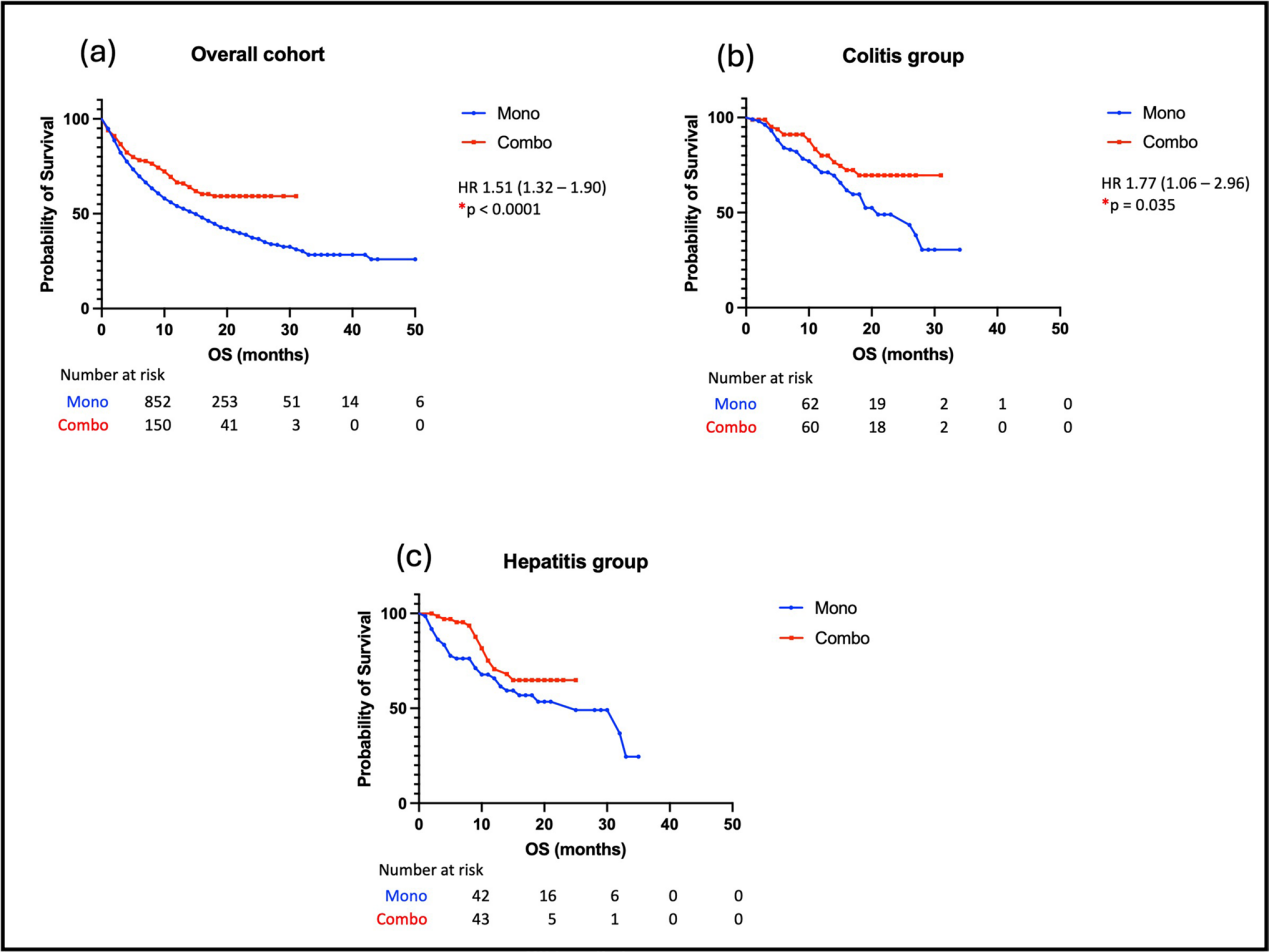
Overall survival (OS) of patients based on treatment subtype—monotherapy (mono) versus combination therapy (combo) (**a**) Patients in the overall cohort; (**b**) Patients with colitis and (**c**) Patients with hepatitis

The median OS in the whole cohort was 8.0 months. Patients who developed colitis had a significantly improved OS (HR 0.42, 95% CI: 0.34 to 0.51, *p* < 0.0001) compared to those who did not develop any irAEs. A similar finding was observed in patients who developed hepatitis (HR 0.50, 95%CI: 0.40 to 0.63, *p* < 0.0001) (see Fig. 1A). The median OS for patients with colitis was 12 months and for those with hepatitis it was 11 months. There was an implied survival benefit for patients treated for MM (HR 0.40, 95% CI:0.32–0.50, *p* < 0.0001) in the overall cohort, but this was not observed when the analysis between different malignancy groups was repeated in the colitis and hepatitis cohorts (see Fig. 1B). Within the cohort of patients treated with cICI there was an improvement in OS observed in the colitis group (HR 0.57, 95% CI: 0.94–0.34, *p* = 0.035), but not in the hepatitis group (see Fig. 2).

### Treatment of GI-irAEs

In the colitis group, a total of 166 (86.9%) patients had details of treatment received recorded. 129 (77.7%) patients received steroids alone and 37 (22.3%) were treated with steroids as well as additional immunosuppressant (IS). The main IS used in this group was the anti-tumor necrosis factor (anti-TNF) agent, infliximab. Three patients received methotrexate as a second-line treatment.

In the hepatitis group, 120 (84.5%) patients had details of treatment recorded and 101 (84.2%) of these received steroids alone whilst 19 (15.8%) also required IS. Only one patient received tacrolimus, whilst the majority (17) received MMF as second line therapy. Three patients who had MMF also went on to subsequently require third line treatment with tacrolimus. Two patients received infliximab as they had colitis concurrently, but they did not receive any specific additional IS for their hepatitis.

Multiple logistic regression was carried out to assess whether there were any predictive factors for the use of second line IS in each patient group. The presence of PAID (HR 4.58, 95%CI: 1.28–16.44, *p* = 0.02) and the use of cICI (HR 10.83, 95% CI: 1.99–58.70, *p* = 0.006) were significant predictors for the use of second line therapy in the colitis group, whereas grade 2 (HR 2.32, 95% CI:1.45–3.65, *p* < 0.001) and 3 (HR 3.21, 95% CI:1.28–6.32, *p* < 0.001) were predictors in the hepatitis group (see Table 5).

**Table 5:** Multivariate logistic regression for association between characteristics and use of additional immunosuppressants

Characteristic	Colitis		Hepatitis	
	**OR (95% CI)**	***p*-value**	**OR (95% CI)**	***p*-value**
Age	0.98 (0.934–1.03)	0.356	1.00 (0.95–1.05)	0.975
Gender	0.70 (0.25–1.96)	0.494	0.81 (0.24–2.78)	0.739
Smoking status	1.49 (0.84–2.65)	0.178	0.94 (0.50–1.75)	0.836
Malignancy (Melanoma)	1.78 (0.43–7.32)	0.424	0.70 (0.10–4.78)	0.719
Maximal grade of G-irAE				
2	0.31 (0.5–1.97)	0.214	2.32 (1.45–3.65)	< 0.001*
3	1.35 (0.24–7.59)	0.734	3.21 (1.28–6.32)	< 0.001*
4	0	0	0	0
PAID	4.58 (1.28–16.44)	0.020*	0.82 (0.08–8.06)	0.862
ICI therapy (cICI vs mICI)	10.83 (1.99–58.70)	0.006*	3.82 (0.76–19.23)	0.104

Abbreviations: PAID, pre-existing autoimmune disease; mICI, monotherapy; cICI, combination therapy

^*^denotes statistical significance at *p* < 0.05

### Outcomes of patients following treatment with IS for GI-irAEs

**Fig. 3:**
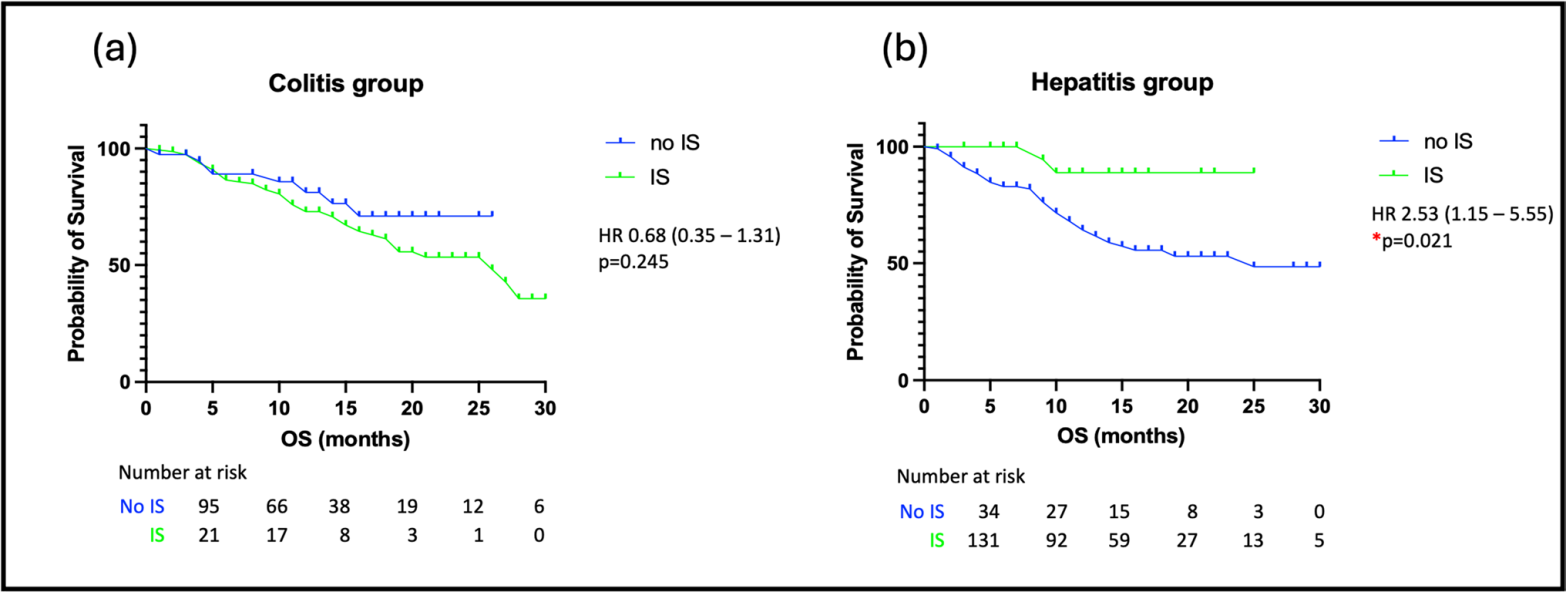
Overall survival (OS) in patient groups when stratified by the use of additional immunosuppressants (IS) (**a**) OS of patients with colitis requiring IS compared to patients not requiring IS and (**b**) OS of patients with hepatitis requiring IS compared to patients not requiring IS

OS was compared between those patients who received second line IS for colitis or hepatitis versus those who had steroids alone. The use of IS did not affect OS significantly in the colitis group (HR 0.65, 95%CI: 0.34–1.24, *p* = 0.245) but was associated with increased survival in the hepatitis group (HR 0.22, 95%CI: 0.14–0.48, *p* = 0.021) (see Fig. 3).

## Discussion

This large multi-centre cohort study reports several key findings relating to ICI-related colitis and hepatitis. These include the identification of several patient risk factors to predict the development of colitis and defined the relationship between Gi-irAEs and overall survival.

The incidences of both colitis and hepatitis experienced in this cohort were similar to those observed in prior clinical trials [13–16]. There was a higher proportion of GI-irAEs observed in the group of patients treated for MM, most likely attributed to the use of cICI in this cancer type. This was also observed in a systematic review by Ma et al. which found that the incidence of colitis and hepatitis was significantly increased in cICI vs mICI [17]. Several phase III clinical trials have also reported an increased risk of colitis with the use of cICI vs mICI in patients with metastatic melanoma, highlighting the additive effects of using two drugs which have different mechanisms of action [18, 19]. It is reassuring to note that the rates of severe colitis and hepatitis in our non-preselected real-world cohort did not differ significantly from those reported in clinical trials.

Previous studies have identified a number of clinical characteristics that potentially predict the development of irAEs. Our findings that PAID and type of ICI used (cICI vs mICI) were risk factors for colitis are consistent with observations in other studies [20, 21]. It is also interesting that the risk factors for developing colitis and hepatitis were different, highlighting the heterogeneity of irAEs. There were only a small number of patients in this cohort who had pre-existing IBD therefore it is difficult to interpret whether it is a risk factor to developing ICI-induced colitis as previously demonstrated in other studies with larger numbers of patients with IBD [22, 23]. A systematic review found that up to 40% of patients with IBD have a relapse in colitis following ICI treatment, in particular with the use of anti- CTLA-4 agents [24]. Whilst pre-existing IBD does not preclude patients from having ICI therapy, these findings suggest that factors such as type of agents used, status of IBD and the patient’s current IBD treatment before initiation of ICI therapy are reviewed prior to commencing treatment.

A review by Wang et al. which analysed risk factors for hepatitis following immunotherapy noted that the melanoma subgroup experienced higher rates of hepatitis than other cancer types, which is consistent with our findings [25]. Several studies have previously reported an association between female sex and hepatitis, but this was not observed in our cohort [26, 27]. Another interesting finding in our study is that younger patients had a higher risk of developing hepatitis. It may therefore be prudent for younger patients to undergo additional monitoring of liver function when starting immunotherapy, particularly those receiving cICI. Cho et al. reported similar findings and also described an increased risk of hepatitis with concomitant use of medications such as acetaminophen and HMG-CoA reductase inhibitors [28]. Unfortunately, we did not collect information about concurrent drug use in our study, but it is certainly an important risk factor to consider as the diagnosis of hepatitis secondary to ICIs is based on excluding other potential causative factors. Insight into these factors, as well as the discovery of other biological factors such as immune signatures and germline genetic variation that modulate susceptibility towards developing irAEs may in the future be used to risk stratify patients for more personalised treatment selection.

Several studies have reported that patients who experience irAEs have a better overall survival than those who do not develop any irAEs [28–30]. This relationship is most likely attributed to the underlying mechanism of action of ICIs. In a study of 327 patients in MD Anderson, diarrhoea was found to be an independent predictor of improved survival regardless of treatment needed [31]. In this study, as well as in our cohort, patients who were treated with cICI and developed colitis had a significantly improved OS when compared to those treated with mICI. A similar trend towards improved OS was also noted in the hepatitis cohort in this study. Interestingly, a recent study investigating the effects of durvalumab, a PD-L1 inhibitor, found that patients who developed a liver injury during treatment had a significantly lower survival than those who did not [32]. We do not have any patients treated with this ICI in our cohort, but this observation highlights the variability present depending on the agent used.

There is currently uncertainty about the most appropriate use of immunosuppressants, particularly second-line immunosuppressants, to treat irAEs. There is a lack of long-term data on drug safety in this patient population, as typically these medications are not utilised in patients with malignancy. It has been postulated that immunosuppressants may counteract the effects of ICIs and cause other adverse events such as an increased susceptibility towards developing infection [33]. Our study demonstrated that the use of IS did not have a negative impact on OS in the colitis group and in fact, in the hepatitis group, demonstrated that patients who were treated with IS had improved OS. The MD Anderson study also found that OS did not differ between patients who were treated with steroids alone compared to those treated with infliximab [31]. This study did also report there was a trend towards increased rates of infection in patients who were treated with steroids without infliximab for a longer duration, suggesting that early treatment with IS should be considered for patients in whom steroids have failed, so that the steroids can be stopped sooner.

There is conflicting evidence surrounding the use of steroids and IS in ICI-induced hepatitis. A systematic review by Peeraphatdit et al. reported that half of patients with grade 3 and 4 ICI-induced hepatitis improved without the need for steroids despite guideline recommendations to start steroids at grade 2 and concluded that the use of steroids could be avoided in some patients with hepatitis [34]. A report by Gauci et al*.* however reported a decreased recovery time for patients treated with steroids versus those who did not receive steroid treatment, but this was not significant due to the small subgroup numbers [35]. This may have an impact if patients are having delays in their ICI therapy due to unresolved hepatitis. In our cohort, the majority of patients received steroid therapy and only 17 (14.2%) required additional IS specifically to treat hepatitis. These patients had a better OS, suggesting that IS are effective and safe to use in steroid refractory cases. A study from Oxford concluded similarly that IS should be considered in cases where transaminitis does not recover within a few days. However the number of patients in this study was small and this finding needs to be validated in larger cohorts [36].

This study presents the results of analysis of a large dataset and has provided novel insights about the incidence, risk factors and outcomes of patients with GI-irAEs following ICI treatment. The findings add to the data available in literature from various other ‘real-world’ cohorts and highlight the heterogeneity of toxicity due to ICIs. There is a need for prospective studies to examine in closer detail the factors highlighted in these studies in order to develop robust risk stratification tools for patients which will lead to better prevention, diagnosis and management of GI-irAEs.

### Limitations

The retrospective design of this study introduces certain limitations, such as selection bias and variability in data availability across different sites. Additionally, we acknowledge that the data presented are historical. However, the findings remain highly relevant as ICIs continue to be used for the same indications in current clinical practice, particularly in the malignant melanoma cohort. It would be prudent to pursue further studies looking at GI-irAEs in the context of novel treatment modalities such as combination ICIs with SACT.

## Data Availability

The datasets generated and analysed during the current study are not publicly available due to information governance restrictions and patient confidentiality requirements.
